# Alcohol Diffusion in Alkali-Metal-Doped Polymeric Membranes for Using in Alkaline Direct Alcohol Fuel Cells

**DOI:** 10.3390/membranes12070666

**Published:** 2022-06-28

**Authors:** Andrea Fernández-Nieto, Sagrario Muñoz, Vicenta María Barragán

**Affiliations:** Department of Structure of Matter, Thermal Physics and Electronics, Faculty of Physics, University Complutense of Madrid, 28040 Madrid, Spain; andreafernandeznieto@gmail.com (A.F.-N.); smsm@ucm.es (S.M.)

**Keywords:** anion exchange membrane, alcohol, doping capacity, crossover, alcohol permeability, alkaline direct alcohol fuel cell

## Abstract

The alcohol permeability of anion exchange membranes is a crucial property when they are used as a solid electrolyte in alkaline direct alcohol fuel cells and electrolyzers. The membrane is the core component to impede the fuel crossover and allows the ionic transport, and it strongly affects the fuel cell performance. The aim of this work is to compare different anion exchange membranes to be used as an electrolyte in alkaline direct alcohol fuels cells. The alcohol permeability of four commercial anion exchange membranes with different structure were analyzed in several hydro-organic media. The membranes were doped using different types of alkaline doping agents (LiOH, NaOH, and KOH) and different conditions to analyze the effect of the treatment on the membrane behavior. Methanol, ethanol, and 1-propanol were analyzed. The study was focused on the diffusive contribution to the alcohol crossover that affects the fuel cell performance. To this purpose, alcohol permeability was determined for various membrane systems. The results show that membrane alcohol permeability is affected by the doping conditions, depending on the effect on the type of membrane and alcohol nature. In general, heterogeneous membranes presented a positive correlation between alcohol permeability and doping capacity, with a lower effect for larger-size alcohols. A definite trend was not observed for homogeneous membranes.

## 1. Introduction

The great environmental damage caused by fossil fuels, coupled with the limited amount of them, has encouraged the research for alternative energy sources [1]. One of the biggest promises in this area is the membrane-based fuel cell technology [2]. This fact has encouraged the development of membranes with the appropriate characteristics to be used in this application [3,4], as well as in the electrolyzers to generate green hydrogen [5,6].

Acid membranes, such as Nafion, are commonly used as polymer electrolyte membranes in fuel cells. However, in comparison to acid proton exchange membrane fuel cells (PEMFCs), the alkaline medium rendered by anion exchange membrane fuel cells (AEMFCs) presents advantages such as the potential to use non-precious-metal catalysts. Moreover, alkaline membrane water electrolysis is a relatively new technology with the advantages of both alkaline water electrolysis (AWE) and proton exchange membrane water electrolysis (PEMWE), overcoming some of their limitations [7]. This technology has been scarcely investigated so far [8]. For this reason, the design of new anion exchange membranes for AEMFC applications has attracted attention, and the number of published articles on membranes for AEMFCs’ applications has increased continuously over the last decade [9,10,11,12].

Among the possible types of fuel cells, direct alcohol fuel cells (DAFCs) emerge as a good candidate for power sources for portable devices and household appliances [13,14,15]. Although methanol has been more extensively explored in DFCs to replace hydrogen fuel because of this higher energy density, other fuels such as ethanol, n-propanol, or ethylene glycol are also possible alternative fuels to hydrogen with high energy density. Moreover, they are easy to store, transport, and handle [16]. Similarly, alcohol electrolysis using polymeric membranes as the electrolyte is a promising route for storing excess renewable energy in hydrogen [17,18]. In portable power applications, alkaline alcohol solution electrolysis can be suitable to obtain hydrogen fast and pure at low temperature [19]. Moreover, alcohol electrolysis reduces the energy demand compared to water electrolysis. The oxidation of the alcohol molecule takes place at lower electrical potential than that required to achieve water splitting [20,21]. Moreover, some results suggest that alcohol electrolysis is more efficient using OH-conducting membranes under appropriate operation conditions [22].

One of the main problems in a direct alcohol fuel cell is the transport of nonoxidized alcohol through the membrane and the dehydration of the typically used Nafion proton exchange membrane [23]. This transport is known as crossover and has two contributions. One is the alcohol diffusion from the anode to the cathode due to the existing concentration gradient, and the other is the electro-osmotic transport accompanying the charge carrier ions [24]. Exploring alkaline fuel cells with anion exchange membranes as the electrolyte to replace cation exchange membranes has been one of the suggested solutions [25]. Unlike in an acid cell, the electro-osmotic transport of alcohol is not an issue in an alkaline fuel cell, since the ionic flow is in this case due to hydroxide ions, and it occurs in the reverse direction to that in proton conduction systems. However, the alcohol diffusion causes conversion losses in terms of lost fuel and depolarization losses at the cathode, strongly affecting the fuel cell performance. The alcohol permeability is also a crucial factor in alkaline-exchange-membrane-based alcohol electrolysis. In this application, the membrane acts as a barrier to the passage of fuel, and its alcohol permeability is a fundamental issue. A common parameter to estimate the diffusion contribution to the crossover is the membrane permeability defined as the product of diffusivity and solubility of the membrane.

In a previous work [26], a correlation was observed between the alkali-doping capacity and the swelling properties of different commercial anion exchange membranes. An effect of the doping process on their alcohol permeability, and so in the diffusion contribution to the alcohol crossover, would be expected when these membranes are used as the electrolyte in direct alcohol fuel cells. The aim of this work was to study this effect for different alcohols and doping agents, and to analyse the influence of the membrane structure. This aspect does not usually receive much attention and it is an important issue as the alcohol crossover is one of the more important factors limiting fuel cell performance.

## 2. Material and Methods

### 2.1. Materials

Four different commercial anion exchange membranes were tested in this study. Ralex AM(H)-PES membrane (hereafter named PES) and AM(H)-PP (hereafter named PP) membrane are composites formed from ion exchange resins with polyethylene basic binder on base quaternary ammonium. Both membranes have different reinforcing material; PES membrane is a polyester-fitting fabric and PP is a polypropylene-fitting fabric. Neosepta AMX membrane (hereafter named AMX) is composed of a styrene divinylbenzene copolymers with tri-alkyl ammonium fixed-charge groups. It contains a reinforcing inert mesh. Fumasep FAP-450 (hereafter named FAP) is a non-reinforced, fluorinated anion exchange membrane. 

With respect to their structure and preparation, Ralex PES and PP membranes are considered as heterogeneous membranes, whereas Neosepta AMX and Fumasep FAP are considered as homogeneous membranes. Membranes were used as received, without any previous treatment. Table 1 shows some of their main properties.

The doping solutions used in this study were selected taking into account the results obtained in a previous work with these same membranes [26]. In that work, it was observed that heterogeneous membrane PP presented the maximum doping capacity, and the homogeneous non-reinforced FAP membrane only presented a significant doping capacity in 1-propanol media. For this reason, the PP membrane was selected for one more completed study, and the doped FAP membranes were only tested in 1-propanol media. The materials used in the experiments were water, methanol (MeOH), ethanol (EtOH), and 1-propanol (1-PrOH) as pure liquids, and water–alcohol mixtures of 1M concentration as solvents. Table 2 shows some properties of the pure liquids and mixtures used as solutions. 

LiOH, NaOH, and KOH of 1M concentration and NaOH of 2M concentration were used as alkaline salts. The alcohol presented in the doping solutions used to dope the membranes with 1M alkaline salts was the same than that used in the diffusion process. Pure pro-analysis-grade chemicals and doubly distilled, degassed pure water were used.

### 2.2. Methods

#### 2.2.1. Membrane Doping

Before the experiments, the membrane samples (Supplementary Materials Figure S1) were dried under vacuum for 24 h and weighted in a high-precision balance (±0.0001 g). After that, the samples were immersed in closed bottles containing the corresponding solution and allowed to equilibrate at controlled temperature by placing the bottles in a large controlled temperature box. After a minimum of seven days of immersion, the swollen membranes were removed from the concentrated alkaline solution and washed in deionized water for several times to remove the free alkali which remained in the membrane. Afterwards, the membranes were dried under vacuum for 24 h and weighted again. The doping capacity, which indicates the amount of alkaline agent retained by the membrane, was estimated from the alkali uptake as [29]:

(1) $$AU\left(\%\right)=\frac{m_{OH-d}-m_{d}}{m_{d}}\times 100$$

where *m_d_* is the mass of the nondoped dry membrane and $m_{OH-d}$ is the mass of the corresponding dry-doped membrane. 

#### 2.2.2. Alcohol Permeability

The membranes were doped as was indicated in the previous section. In this case, after the membranes were removed from the solution and washed in deionized water, they were kept in closed glass containers with deionized water until used. 

**Figure 1:**
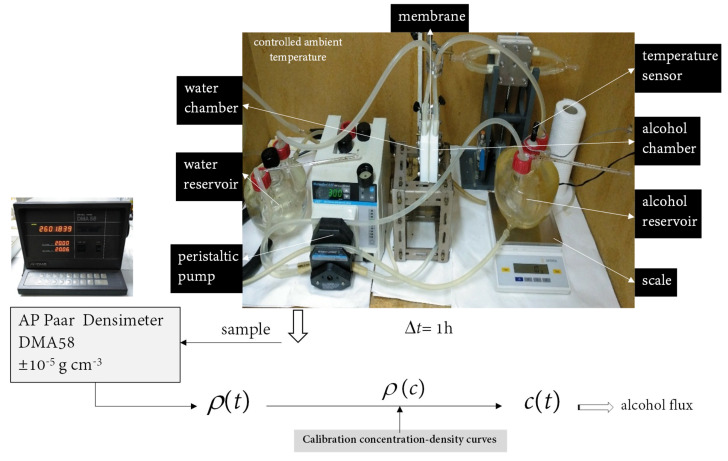
Experimental device used to estimate membrane alcohol permeability.

The experimental device used to measure the alcohol permeability of the membranes, shown in Figure 1, was similar to the one used in previous works [30]. It consisted of a diffusion cell with the membrane separating two chambers, the water chamber containing initially pure water, and the alcohol chamber containing initially a water–alcohol mixture of 50% wt. concentration. Two glass reservoirs of capacity 0.5 × 10^−3^ m^3^ contained the circulation solutions in both chambers. The corresponding solutions circulated from the thermostated reservoirs by means of a peristaltic pump. The circulation velocity of the solutions was set to 300 mL min^−1^. The whole system was immersed in a large, controlled-ambient-temperature box. The temperature of the experiments was 25 °C. The effective area was 18.5 × 10^−4^ m^2^. Pure water was introduced in one reservoir and a water–alcohol solution in the other one. When the temperature of 25 °C was achieved in both chambers, the solutions were circulated through the cell. 

With methanol, a test was also carried out using pure alcohol as initial solutions in the alcohol chamber.

In this device, with the membrane separating two solutions of different concentrations, the alcohol permeability can be modelled on the basis of Fick’s law for a diaphragm–cell diffusion if the experimental conditions allow the assumption of a pseudo-steady state and negligible-concentration polarization effects; that is, a large volume of solution comparing to the membrane volume and well-stirred solutions. The volume of solutions in the experiments was about 3 × 10^−4^ m^3^ in each chamber. 

If we do not consider the existence of water transport through the membrane, the total flux will be only due to the alcohol diffusion and it can be estimated from the change with time of the concentration in one of the chambers, *c*_1_, of the diffusion cell [31].

(2) $$J_{alcohol}=\frac{dc_{1}}{dt}\frac{V_{1}}{A}$$

where *V*_1_ is the volume of the chamber 1 and *A* is the effective membrane area. 

Under pseudo-steady conditions, the concentration change can be considered linear with time, and we can use to estimate the alcohol permeability the known following expression [30,32]:

(3) $$P=\frac{dc_{1}}{dt}\frac{V_{1}^{0}}{A\left(c_{2}^{0}-c_{1}^{0}\right)}$$

where $V_{1}^{0}$ is the initial volume of the water chamber and $c_{1}^{0}$ and $c_{2}^{0}$ are, respectively, the initial concentrations in the water and alcohol chambers. In this case, the concentration was measured in the diluted chamber containing pure water at the initial moment of the process; thus, $c_{1}^{0}=0$. The alcohol permeability is obtained as:

(4) $$P({\mathrm{ms}}^{-1})=\alpha \frac{V_{1}^{0}}{Ac_{2}^{0}}$$

in which parameter *α* indicates the rate of change of alcohol concentration in the corresponding chamber. This is one of the most usual methods to determine ex situ the alcohol permeability of a membrane [29,30,31,32,33,34].

For determining the parameter *α* in our device, the density of the chamber that initially contained pure water was measured every hour during the experiments. We took small solution samples from the water reservoir every hour during six or seven hours of each experiment. The temperature of these samples was led to 20 °C; then, the density measurements were made using an AP Paar Density Meter model MDA58 with an accuracy of ±10^−2^ kcm^−3^. Afterwards, we determined the concentration of the samples as a function of time using previously obtained calibration concentration–density curves [27,28,35,36,37]. For each experiment, the values were fitted to a straight line, whose slope let us calculate the value of parameter *α*. This parameter was used to calculate the permeability by means of Equation (4).

## 3. Results and Discussion

### 3.1. Alcohol Permeability for Nondoped Membranes 

We carried out a previous study with the nondoped membranes to compare them with those subsequently obtained with doped membranes. To this purpose, experiments were carried out with pure methanol and 50% wt. alcohol–water mixtures, using methanol, ethanol, and 1-propanol as alcohols, in the concentrated chamber. 

**Figure 2:**
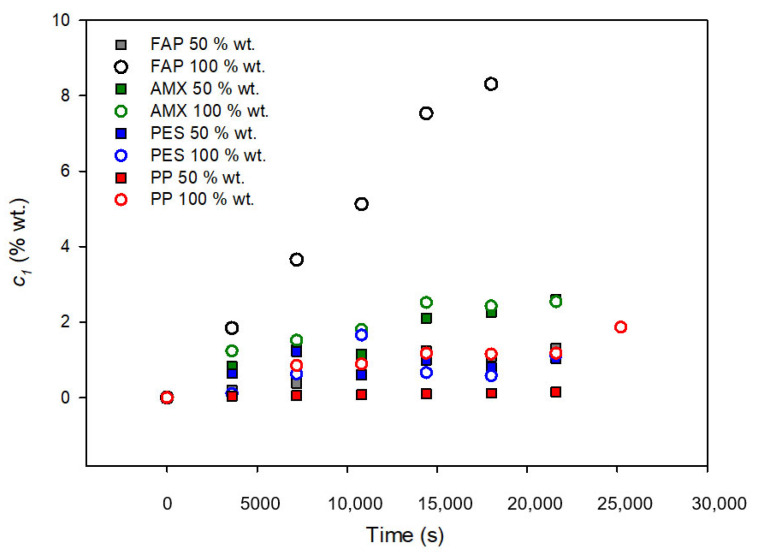
Concentration as a function of time in the water chamber with non-doped membranes with pure methanol and 50% wt. water–methanol mixture in the alcohol chamber.

As an example, the results for the time dependence of the concentration in the water chamber are shown in Figure 2 in the case of using methanol. We can observe that the alcohol concentration always increased in the diluted chamber, according to the diffusion process occurring from the concentrated to the diluted chamber. 

We observed that all the membranes looked, in general, deteriorated at the end of the experiments when pure methanol was used in the concentrated chamber. For this reason, no experiments were carried out with the other pure alcohols, and only 50% wt. water–alcohol mixtures were used as concentrated solutions in the rest of the experiments. Similar concentration–time curves were obtained for nondoped membranes using ethanol and 1-propanol. Different studies suggest that the molecular transport of organic solvent in a rubbery polymer system is controlled by a combination of sorption, diffusion, and permeation mechanisms [38]. With linear alcohols, the polymer matrix is usually unaffected by the diffusant so that diffusion is expected to follow Fick´s law [39], in agreement with the observed behaviour in the experiments.

Table 3 shows the alcohol permeability estimated for the nondoped membranes from the obtained values of parameter *α* and Equation (4) under the different studied conditions.

For nondoped membranes, we can see that larger methanol permeabilities and higher influence of the methanol concentration were observed for homogeneous AMX and FAP membranes. Methanol permeabilities were found to be of similar values to those obtained for other membranes studied in the literature [31,33,40,41]. When we compare the alcohol permeabilities values obtained for different alcohols using a 50% wt. water–alcohol mixture, we can observe that the alcohol permeability decreases with the molar mass of the alcohol for all membranes. This is probably due to the hydrated molecular size increasing with increasing alcohol molar mass, leading to a relatively high resistance for alcohol across the membrane. A similar trend has been also observed with other kinds of membranes [40,41,42]. The highest influence of the membrane structure was observed with methanol. For ethanol, similar values were estimated for all the tested membranes. With 1-propanol, only the non-reinforced homogeneous FAP membrane presented a significant difference with respect to the other membranes. 

### 3.2. Alcohol Permeability for Doped Membranes 

**Figure 3:**
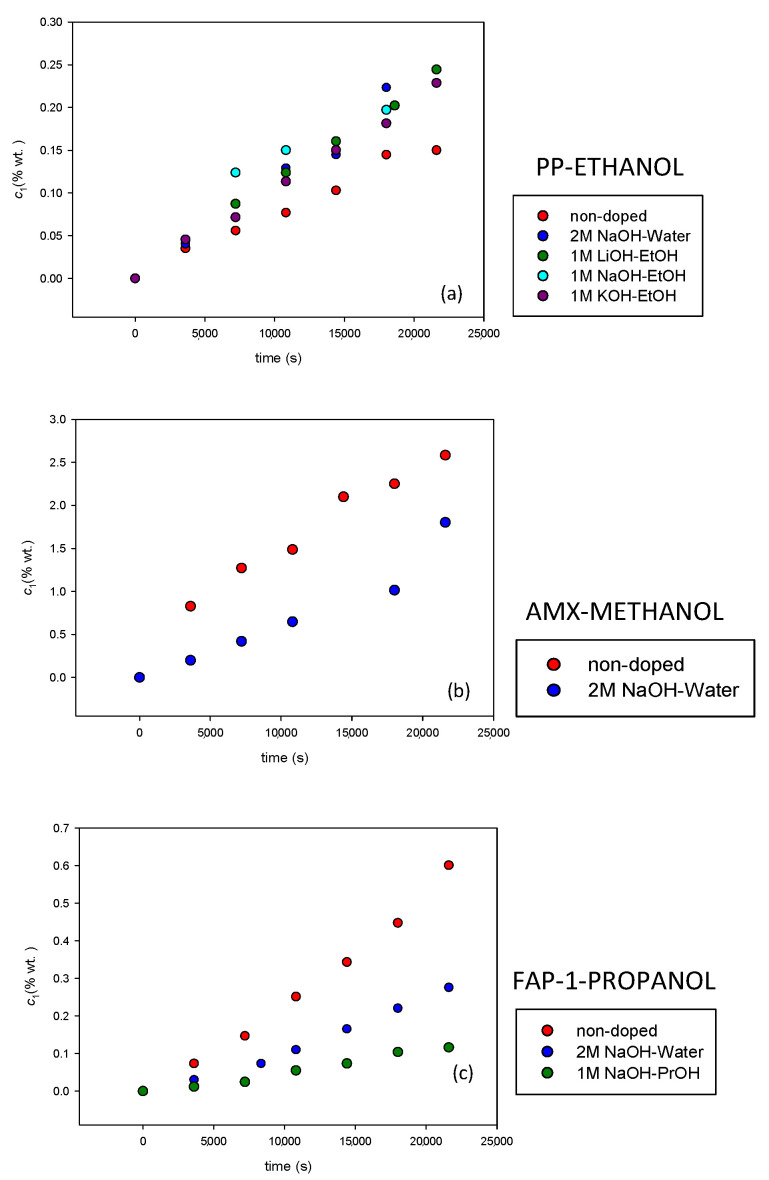
Concentration as a function of time for different membrane systems. (**a**) Heterogeneous PP membrane with ethanol doped with different doping agent in ethanol media. (**b**) Homogeneous AMX membrane with methanol. (**c**) Homogeneous FAP membrane with different doping agent in 1-propanol media.

In Figure 3, examples of the concentration in the water chamber as a function of time for different doped membrane systems are shown. We also included the values of the nondoped membranes in these figures for a better comparison. It can be observed that the behavior was similar to those obtained with the nondoped membranes, with an increase in concentration in the diluted chamber. The more interesting aspect in these figures is to observe that the membrane doping affects the membranes’ alcohol diffusion properties. 

As in the case of the nondoped membranes, from the concentration–time data, parameter *α* was estimated and the value of the alcohol permeability obtained using Equation (4). Table 4 and Table 5 show the results obtained for the doped membranes. The values corresponding to the nondoped membranes were also included for a better comparison. Table 4 shows the values corresponding to the alcohol permeability of membrane PP with the different doping agents.

Table 5 shows the values of the alcohol permeability obtained for doped PES, AMX, and FAP membranes.

The results presented in Table 4 and Table 5 show that the effect of the doping process on the alcohol permeability is different for each membrane and it depends on the doping agent.

**Figure 4:**
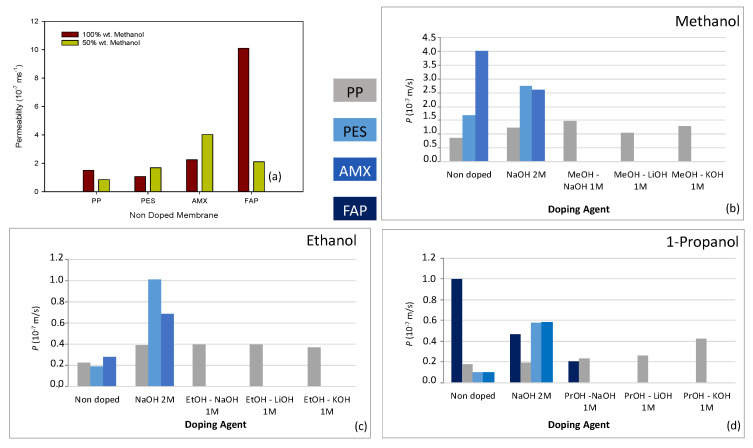
Values of the alcohol permeability estimated for all the membranes investigated in this work.

For a better look of the doping effect on the diffusion properties of the membrane, Figure 4 shows all the alcohol permeability estimates for the different membrane systems analysed. 

Heterogeneous nondoped membranes presented in general lower alcohol permeability values. Nevertheless, when these membranes were alkali-metal-doped, their alcohol permeability increased. This effect was more pronounced for the PES membrane. However, the heterogeneous-doped PP membrane also presented, in general, lower values for the alcohol permeability than the homogeneous membranes, not showing a high influence of the doping agent on the alcohol permeability. The homogeneous AMX membrane also increased its alcohol permeability value after the doping process, with the exception of the sample doped with NaOH 2M, which reduced its methanol permeability. For the homogeneous FAP membrane, the doping process reduced the 1-propanol permeability, mainly when the membrane was doped in 1-propanol medium. 

### 3.3. Alcohol Permeability and Doping Capacity

**Figure 5:**
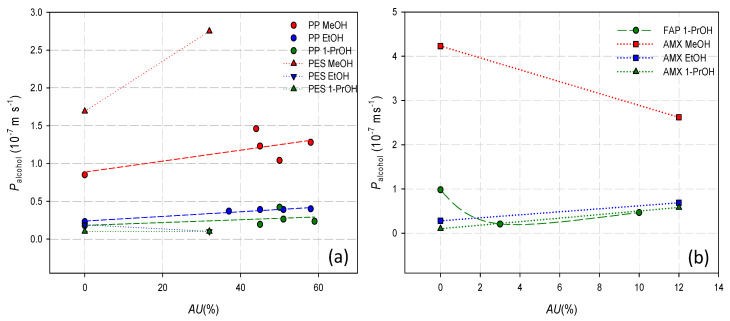
Alcohol permeability as a function of the doping capacity. (**a**) Heterogeneous membranes. (**b**) Homogeneous membranes.

Figure 5a shows the alcohol permeability as a function of the doping capacity for heterogeneous PP and PES membranes. For these membranes, a general increasing trend of the alcohol permeability with the doping capacity was observed. Previous results [26] showed that, for heterogeneous membranes, the doping process led to minor liquid uptakes in the doped membranes and to larger water affinities. Thus, the results would indicate that for the heterogeneous membranes, a lower water content favours the diffusion of alcohol through the membrane. Nevertheless, this effect decreases with increasing the viscosity of the alcohol. 

These results seem to indicate that a key factor in the alcohol permeability of the more porous heterogeneous membranes is the membrane water content. The doping process affects the swelling properties of the membranes and, thus, their diffusive behaviour. Similar results have been found with alkaline-doped PBI membranes. However, for these membranes, the observed increase was explained taking into account the water uptake increase accompanied by the increased alkali uptake. In this case, the increased permeability resulted from the reduced intermolecular interaction due to the established ionic channels [29]. 

For homogeneous membranes, with lower doping capacity and water content, a definite trend was not observed (Figure 5b). Despite their low doping capacity, these membranes showed a great influence of the doping process on their alcohol permeability. It was observed in a previous work [26] that the doping process had low influence on the membrane water uptake. Thus, for denser homogeneous membranes, it cannot be the cause of the observed decrease in the alcohol permeability. The permeability obtained for Equation (4) results from the influence of the membrane thickness. As the doping capacity affects the expansion properties of the membranes, it is possible that the doping process could affect the membrane thickness and, thus, the alcohol permeability. For homogeneous membranes, it was observed that the presence of hydroxide in the solution had, in general, less effect on the membrane surface expansion [26]. It could indicate that the doping effect occurs mainly in the direction of membrane thickness. Further works would be necessary to clarify this statement. 

## 4. Conclusions

The alkali-metal-doping effect on the alcohol diffusion properties of different anion exchange membranes was investigated. The membranes and the doping agents used to carry out the study were selected taking into account previous results about the membranes’ doping capacity. Membranes with higher doping capacity were selected to carry out the study.

Alcohol permeability values of the order of 10^−7^–10^−8^ ms^−1^ were estimated. No definite trend was observed in general between doping agent and permeability, depending on the particular influence of the membrane and type of alcohol. However, all the membranes showed an alcohol permeability decrease with increasing alcohol molar mass, with the trend: *P*_1-PrOH_ < *P*_EtOH_ < *P*_MeOH_, independently of the membrane-doping process.

In general, heterogeneous membranes presented a positive correlation between alcohol permeability and doping capacity, but with a lower effect for larger-size alcohols. This may be due to the doping process causing a decrease in the membrane water content, favouring the alcohol diffusion through the membrane. A definite trend was not observed for homogeneous membranes.

The results obtained show that the doping process affects the diffusion properties of the membranes. However, in general, it increases the membrane alcohol permeability; therefore, it would favour the alcohol diffusion process. Only homogenous membranes showed a decrease in the alcohol permeability after the doping process: the membrane AMX doped with NaOH 2M, which reduced its methanol permeability, and the non-reinforced FAP membrane, which showed a decrease in the 1-propanol diffusion after the doping process.

Only in two of the studied cases would the doping process be useful to reduce the diffusion contribution to the alcohol crossover in DAFCs. This is an important issue to take into account when doping processes are used to modify membranes as the membrane alcohol crossover is one of the more important aspects limiting fuel cell performance.

## Figures and Tables

**Table 1:** Some properties of the used membranes. Thickness (*d*), ion exchange capacity (IEC), density (*ρ*), and electric resistance (*R*).

Membrane	*d* (μm) *	IEC (meq g^−1^) *	Selectivity *	*R* (Ω cm^2^) *	*ρ* (kg m^−^^3^) **
PES	450	1.8	>0.95	<7.5	945
PP	440	1.8	>0.95	<8.0	917
AMX	140	1.5	≥0.98	<3.5	1090
FAP	50	1.2	>0.92	<1.5	1132

* Provided by the manufacturer. ** Measured (See Supplementary Materials).

**Table 2:** Density (*ρ*), viscosity (*ν*), and molar mass (*M*) of the used pure liquid, and density (*ρ*) and viscosity (*ν*) of the used water–alcohol mixtures at 303 K.

Pure Liquid		*ρ* (kg m^−^^3^) *	ν* (mPa s)	*M* (10^−3^ kg mol^−^^1^)
Water	H_2_O	995.7	0.797	18.0
MeOH	CH_4_O	782.0	0.508	32.04
EtOH	C_2_H_6_O	781.3	0.987	46.07
1-PrOH	C_3_H_8_O	796.4	1.726	60.09
**Water–Alcohol Mixture**		***ρ*** **(kg m** **^−^** **^3^** ) *	***ν*** * **(** **mPa s)**	
1M	MeOH	999.2	0.756	
	EtOH	976.4	0.709	
	1-PrOH	990.2	0.920	

* [27,28].

**Table 3:** Alcohol permeability values for nondoped membranes with 100% and 50% wt. alcohol in the alcohol chamber.

*P* (10^−8^ ms^−1^)				
	PP	PES	AMX	FAP
MeOH 100%	15.0 ± 0.1	10.6 ± 0.1	22 ± 4	101 ± 8
MeOH 50%	8.3 ± 0.1	16.7 ± 0.1	42.1 ± 0.1	20.9 ± 0.1
EtOH 50%	2.27 ± 0.14	1.88 ± 0.14	2.82 ± 0.15	--
1-PrOH 50 %	1.82 ± 0.14	1.01 ± 0.18	1.02 ± 0.18	9.7 ± 0.4

**Table 4:** Alcohol permeability values for nondoped PP membrane and doped under different conditions.

*P* (10^−8^ ms^−1^)			
PP	MeOH	EtOH	1-PrOH
Nondoped	8.3 ± 0.1	2.27 ± 0.15	1.82 ± 0.14
NaOH 2M Water	12.1 ± 0.1	3.9 ± 0.3	1.93 ± 0.07
LiOH 1M	10.3 ± 0.2	3.92 ± 0.07	2.62 ± 0.25
NaOH 1M	14.4 ± 0.1	4.00 ± 0.07	2.31 ± 0.25
KOH 1M	12.6 ± 0.2	3.68 ± 0.07	4.17 ± 0.20

**Table 5:** Alcohol permeability values for PES, AMX, and FAP membranes, nondoped and doped with different doping agents.

*P* (10^−8^ ms^−1^)			
	MeOH	EtOH	1-PrOH
PES			
Nondoped	16.7 ± 0.1	1.88 ± 0.14	1.01 ± 0.18
NaOH 2M Water	27.3 ± 0.3	10.1 ± 0.3	5.8 ± 0.3
**AMX**			
Nondoped	42.1 ± 0.3	2.82 ± 0.15	1.02 ± 0.18
NaOH 2M Water	26.0 ± 0.3	6.83 ± 0.14	5.78 ± 0.18
**FAP**			
Nondoped	20.9 ± 0.1	--	9.7 ± 0.4
NaOH 2M Water	--	--	4.65 ± 0.25
NaOH 1M	--	--	2.05 ± 0.24

## Data Availability

Not applicable.

## Supplementary Materials

The following are available online at https://www.mdpi.com/article/10.3390/membranes12070666/s1, Figure S1: Membrane sample.
