# Supplementary material for: Alcohol Diffusion in Alkali-Metal-Doped Polymeric Membranes for Using in Alkaline Direct Alcohol Fuel Cells
Source: Membranes (Basel). 2022 Jun 28;12(7):666. doi: 10.3390/membranes12070666 (PMC9318451; doi:10.3390/membranes12070666)
Supplement: Supplementary file 1 [file membranes-12-00666-s001.zip › membranes-1767942-supplementary.pdf]

$l_x$

Supplementary Material for

# Alcohol diffusion in alkali-metal-doped polymeric membranes for using in alkaline direct alcohol fuel cells.

A. Fernández-Nieto, S. Muñoz, V. M. Barragán*

*Department of Structure of Matter, Thermal Physics and Electronics, Faculty of Physics, University Complutense of Madrid, 28040 Madrid, Spain.*

## Estimation of the membrane density

The membrane density was estimated according to the expression:

$$\rho = \frac{m}{V} \quad \text{(S1)}$$

where *m* and *V* are, respectively, mass and volume of the membrane sample.

Samples of surface approximately square about 100x100 mm$^2$ were used (Fig. S1). The volume was estimated as:

$$V = l_x l_y d \quad \text{(S2)}$$

where $l_x$ and $l_y$ are the two sides of the membrane sample, and *d* is the membrane thickness.

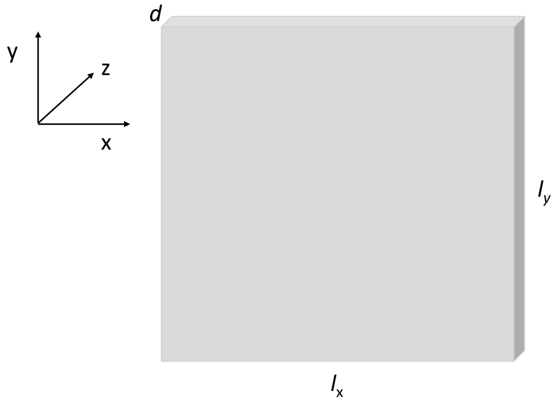


**Figure S1**. Membrane sample

The membrane samples were dried under vacuum for 24 h and weighted in a high precision balance (±0.0001g). Afterwards, the length of the two sides of the squared sample was measured with a palmer (±0.01mm), and the membrane thickness was measured with a PCE-THM-20 material thickness meter with resolution ±0.0002 mm. Final value of membrane thickness was obtained by averaging the results of at least ten measurements made at different points of the sample under study. The maximum standard error was always lower than 0.001 mm.
